# Saikosaponin d induces cell death through caspase-3-dependent, caspase-3-independent and mitochondrial pathways in mammalian hepatic stellate cells

**DOI:** 10.1186/s12885-016-2599-0

**Published:** 2016-07-26

**Authors:** Ming-Feng Chen, S. Joseph Huang, Chao-Cheng Huang, Pei-Shan Liu, Kun-I Lin, Ching-Wen Liu, Wen-Chuan Hsieh, Li-Yen Shiu, Chang-Han Chen

**Affiliations:** 1 Department of Gastroenterology and Hepatology, E-DA Hospital, Kaohsiung, Taiwan; 2 Graduate Institute of Integrated Medicine, China Medical University, Taichung, Taiwan; 3 Department of Obstetrics and Gynecology, E-Da Hospital, I-Shou University, Kaohsiung, Taiwan; 4 Department of Obstetrics and Gynecology, University of South Florida, College of Medicine, Tampa, FL USA; 5 Department of Pathology, Kaohsiung Chang Gung Memorial Hospital, Chang Gung University College of Medicine, Kaohsiung, Taiwan; 6 Tissue Bank and Biobank, Kaohsiung Chang Gung Memorial Hospital, Kaohsiung, Taiwan; 7 Department of Microbiology, Soochow University, Shihlin, Taipei Taiwan; 8 Departments of Obstetrics & Gynecology, Chang Bing Show Chwan Memorial Hospital, Lukang Zhen, Changhua County Taiwan; 9 School of Pharmacy, Kaohsiung Medical University, Kaohsiung, Taiwan; 10 Department of Biological Science & Technology, I-SHOU University, Kaohsiung, Taiwan; 11 Department of Medical Research, E-Da Hospital, Kaohsiung, Taiwan; 12 Department of Medical Research, Cell Therapy and Research Center, E-Da Hospital, I-Shou University, No.6, Yida Road, Jiaosu Village, Yanchao District, Kaohsiung City, 82445 Taiwan People’s Republic of China; 13 Institute for Translational Research in Biomedicine, Kaohsiung Chang Gung Memorial Hospital, 123 Ta-Pei Road, Niaosong District, Kaohsiung City, Taiwan People’s Republic of China; 14 Department of Applied Chemistry, National Chi Nan University, Nantou, Taiwan; 15 Center for Infectious Disease and Cancer Research, Kaohsiung Medical University, Kaohsiung, Taiwan

## Abstract

**Background:**

Saikosaponin d (SSd) is one of the main active triterpene saponins in *Bupleurum falcatum*. It has a steroid-like structure, and is reported to have pharmacological activities, including liver protection in rat, cell cycle arrest and apoptosis induction in several cancer cell lines. However, the biological functions and molecular mechanisms of mammalian cells under SSd treatment are still unclear.

**Methods:**

The cytotoxicity and apoptosis of hepatic stellate cells (HSCs) upon SSd treatment were discovered by MTT assay, colony formation assay and flow cytometry. The collage I/III, caspase activity and apoptotic related genes were examined by quantitative PCR, Western blotting, immunofluorescence and ELISA. The mitochondrial functions were monitored by flow cytometry, MitoTracker staining, ATP production and XF24 bioenergetic assay.

**Results:**

This study found that SSd triggers cell death via an apoptosis path. An example of this path might be typical apoptotic morphology, increased sub-G1 phase cell population, inhibition of cell proliferation and activation of caspase-3 and caspase-9. However, the apoptotic effects induced by SSd are partially blocked by the caspase-3 inhibitor, Z-DEVD-FMK, suggesting that SSd may trigger both HSC-T6 and LX-2 cell apoptosis through caspase-3-dependent and independent pathways. We also found that SSd can trigger BAX and BAK translocation from the cytosol to the mitochondria, resulting in mitochondrial function inhibition, membrane potential disruption. Finally, SSd also increases the release of apoptotic factors.

**Conclusions:**

The overall analytical data indicate that SSd-elicited cell death may occur through caspase-3-dependent, caspase-3-independent and mitochondrial pathways in mammalian HSCs, and thus can delay the formation of liver fibrosis by reducing the level of HSCs.

## Background

Hpatic stellate cells (HSCs) play important roles in vitamin A metabolism and extracellular matrix (ECM) production. During liver injury progression, HSCs may be activated directly or indirectly by cytokines or reactive oxygen species (ROS) released from injured cells. These microenviromental activations trigger quiescent HSCs to undergo phenotypical transformation and develop a myofibroblast-like phenotype. Activated HSCs produce type I and III collagen; express α-smooth muscle actin (α-SMA), and display a high proliferation rate, ECM synthesis, chemotaxis and cytokine release [1, 2]. Most studies on HSCs focus on proliferation inhibition and apoptosis induction, and some on cell migration and relevant mechanisms.

*Bupleurum falcatum* has been used in traditional Chinese medicine to treat liver injury for thousands of years. As the major active component of triterpene saponin in *Bupleurum falcatum*, SSd has a common steroid-like structure, and is reported to have pharmacological activities [3–7]. In particular, accumulating evidence has indicated that SSd could protect against CCl_4_- and dimethylnitrosamine-induced liver injury in rats [5, 8, 9]. Recent studies have indicated that SSd induces cell cycle arrest and apoptosis in several cancer cell lines via modulating following factors, including p53, nuclear factor kappa B and Fas/Fas ligands [10–14]. Moreover, SSd promotes apoptosis and G1-phase cell cycle arrest in undifferentiated thyroid carcinoma through the up-regulation of p53, BAX and p21, and down-regulation of Bcl-2, CDK2 and cyclin D1 expression [15]. SSd also sensitizes cancer cells to cisplatin through ROS-mediated apoptosis, and prevents carcinogen-induced tumorigenesis [16]. Our previous report showed that SSd inhibited the proliferation of HSC-T6 cells, wound healing and cell migration. Additionally, SSd triggers HSC-T6 apoptosis, and blocks platelet-derived growth factor (PDGF)-BB- and tumor growth factor (TGF)-β1-induced cell proliferation and migration [17]. However, the precise mechanisms underlying SSd-induced HSC apoptosis are still not clear.

This study elucidates the mechanism underlying SSd-induced cell death in HSCs via caspase-dependent and caspase-independent pathways. The role of mitochondrial fractures in apoptosis is also examined.

## Method

### Cell culture and cell proliferation assay

A human HSC cell line, LX-2 was purchased from MERCK MILLIPORE (SCC064). A rat HSC cell line, HSC-T6, immortalized with the large T antigen of the SV40. Both cell lines were cultured at 37 °C under a 5 % CO_2_ atmosphere and in Dulbecco’s modified Eagle’s medium (DMEM; Gibco®, Life Technologies) supplemented with 100U/mL penicillin, 100 μg/mL streptomycin and 5 % heat-inactivated fetal bovine serum (FBS; Gibco®, Life Technologies). The HSC-T6 and LX-2 cells were seeded in 96-well plates at a density of 5 × 10^3^ cells/well in DMEM supplemented with 5 % FBS. Following an 18-h incubation at 37 °C under a 5 % CO_2_ atmosphere, the old medium was replaced with fresh DMEM/1%FBS containing SSd (1 μM). After 0, 18, 24, 48 and 72 h of incubation, the 3-(4,5-dimethylthiazol-2-yl)-2,5-diphenyltetrazolium bromide (MTT) assay (Invitrogen™, Life Technologies) was performed for cell proliferation detection as describe previous report [18]. The generated formazan products were solubilized with 100 μL of dimethyl sulfoxide (DMSO; Sigma-Aldrich), and the optical density was determined at 570 nm using an enzyme-linked immunosorbent (ELISA) reader (infinite M200PRO, TECAN).

### Colony formation assay

Colony formation assay was performed according to Park *et al.*’s work [19, 20]. Both HSC-T6 and LX-2 cells were seeded (2 × 10^3^ cells/well) in a 6-well plate and incubated at 37 °C under a 5 % CO_2_ atmosphere for 14 days. The colonies were fixed with 70 % ethanol at 4 °C and stained with 5 % Gentian Violet (Sigma) at room temperature.

### Detection of apoptosis

Specific apoptosis was evaluated in both HSC-T6 and LX-2 cells by treating with SSd (1 μM) for 24 h. All living cells and cell debris were collected and fixed in 70 % ethanol/phosphate-buffered saline (PBS) at 4 °C, pelleted and resuspended in a buffer solution containing 200 μg/mL RNase A and 0.01 mg/mL propidium iodide (PI; Sigma-Aldrich). The cell cycle states of the HSC-T6 and LX-2 cells were determined by flow cytometry (Cytomic FC 500, BECKMAN COULTER).

### Protein detection of collagen, caspase-3/7 and caspase-9

Both HSC-T6 and LX-2 cells were seeded in a 6-well culture plate at a density of 2 × 10^5^ cells/well to determine the protein expression levels of collagen type I, collagen type III, caspase-3 and caspase-9. After 16-h incubation, the exhausted culture medium was discarded and replaced with fresh serum-free DMEM (Gibco®, Life Technologies) containing SSd at a working concentration of 1 μM). The culture medium and living cells were collected after subsequent 24-h incubation. The cell pellet was lysed by RIPA solution, and the total protein content was extracted. The protein expressions of collagen type I and III, caspase-3, and caspase-9 were measured by ELISA kits (Uscn Life Science Inc.). The activity of caspase-3/7 and caspase-9 was measured by the ApoTox-Glo™ Triplex assay kit (for caspase-3/7 activity detection; Promega) and Caspase-Glo® 9 assay kit (for caspase-9 activity detection; Promega) according to the manufacturer’s (protocol TRY instructions). The ApoTox-Glo™ Triplex assay kit also provides information on apoptosis. HSC-T6 and LX-2 cells were treated by SSd (0 μM, 0.1 μM, 0.25 μM, 0.5 μM, 1 μM, 1.5 μM, and 2 μM) for 24 h, and the results from 3 reactions at each SSd concentration were averaged. The fluorescence was measured by an ELISA reader (Fluoroskan Ascent FL, THERMO SCIENTIFIC), and the luminescence was measured by a luminometer (Centro LB 960, CBERTHOLD TECHNOLOGIES).

### RNA isolation and quantitative RT-PCR

Total RNA was isolated by TriPure Isolation Reagent (Roche) based on the manufacturer’s protocol. Reverse transcriptional PCR was performed using the iScripe™ cDNA Synthesis kit (BIO-RAD). Quantitative polymerase chain reaction (qPCR) analysis and data collection were performed by an ABI 7500 FAST (Applied Biosystem). The following sequences of specific primers of target genes were adopted in qPCR: BAD-forward, 5′-CAGGCAGCCAATAACAGTCATC-3′; BAD-reverse, 5′-CCATCCCTTCATCTTCCTCAGT-3'; BAK-forward, 5′-AATGCCTACGAACTCTTCACCAA-3'; BAK-reverse, 5′-CAGTCAAACCACGCTGGTAGAC-3′; BAX-forward, 5′-TCATCCAGGATCGAGCAGAGA-3′; BAX-reverse, 5′-CCAATTCGCCGGAGACACT-3′; Bcl-2-forward, 5′-CATCTGCACACCTGGATCCA-3′; Bcl-2-reverse, 5′-TGAGCAGCGTCTTCAGAGACA-3′; Bcl-xL-forward, 5′-GATGGCCACCTACCTGAATGA-3′; Bcl-xL-reverse, 5′-CTCGGCTGCTGCATTGTTC-3′; PUMA-forward, 5′-ATGGCGGACGACCTCAAC-3'; PUMA-reverse, 5′-GGGAGGAGTCCCATGAAGAGA-3′.

### Mitochondrial and cytosolic fractions isolation and protein detection

HSC-T6 cells (1 × 10^7^ cells) were treated with SSd (1 μM) for 0, 0.25, 0.5, 1, 2, 4, and 8 h. Their mitochondrial and cytosolic fractions were then isolated by a Mitochondria/Cytosol Fractionation Kit (BioVision). Protein expression was detected by Western blotting using specific antibodies. The protein levels of COX3, GAPDH, BAX, BAK, apoptotic-protease-activating factor (Apaf)-1, cytochrome c (Cyt c), endonuclease G (EndoG) and apoptosis-inducing factor (AIF) were determined in isolated mitochondrial and cytosolic fractions (25 μg).

### Western blotting

The cells were subsequently lysed in RIPA solution containing protease inhibitors (Roche). The total extracted protein content (25 μg) was separated by SDS-PAGE and transferred to polyvinyl difluoride (PVDF) membranes. The PVDF membranes were incubated by primary antibodies at a dilution of 1:500 or 1:1000 to detect procaspase-9, caspase-9, procaspase-3, caspase-3 (Cell Signalling), COX3, GAPDH (Santa Cruz), collagen I, collagen III, BAX, BAK, Bcl-2, Bcl-xL, Bcl-2-associated death promoter (BAD), p53 upregulated modulator of apoptosis (PUMA), β-actin, Apaf-1, Cyt c, EndoG, AIF (GeneTex) and α-SMA (abcam). The fold change in protein expression was expressed as a ratio calculated by dividing the specific protein band density by the β-actin band density.

### Mitochondrial membrane potential change measurement and mitochondrial staining

The mitochondrial membrane potential (∆*ψ*m) during apoptosis was monitored by a MitoProbe JC-1 assay kit (Molecular Probe), which is a lipophilic cationic dye. In brief, HSC-T6 cells were exposed to SSd (1 μM) for 30 and 60 min, and the cells were subsequently labeled with 2 μM JC-1 dye for 30 min at 37 °C. All cells were collected, washed twice with PBS, and analyzed by flow cytometry (Cytomic FC 500, BECKMAN COULTER). For mitochondrial staining, HSC-T6 cells were grown on coverslips for 16 h. After treatment with 1 μM SSd for 0, 15, 30 and 60 min, mitochondria were stained with 100nM MitoTracker® Deep Red FM (Invitrogen™, Life Technologies) for 30 min at 37 °C. DAPI (Molecular Probe) was adopted as a nuclear counterstain, and images were acquired by a confocal laser-scanning microscope (TCS SP5, LEICA).

### ATP production, oxygen consumption, and extracellular acidification detection

Cellular ATP levels were detected by the Mitochondrial ToxGlo assay (Promega) according to the manufacturer’s protocol. Briefly, HSC-T6 cells were cultured at 1 × 10^4^ cells/well in a white, clear-bottom 96-well culture plate. The culture was incubated for 16 h, then the exhausted medium was discarded and replaced with a fresh medium containing 10 mM galactose instead of glucose. The cells were subsequently treated with SSd (0 μM, 0.1 μM, 0.25 μM, 0.5 μM, 0.75 μM, 1 μM, 1.5 μM, and 2 μM) for 24 h. An equal volume of the assay solution was added to each well, and the plate was subsequently incubated at room temperature for 30 min. Luminescence was measured by a luminometer (Centro LB 960, CBERTHOLD TECHNOLOGIES). The cellular oxygen consumption rate (OCR) and extracellular acidification rate (ECAR) were measured by an XF24 bioenergetic assay (Seahorse Bioscience, Billerica, MA). Briefly, HSC-T6 cells were suspended in DMEM containing 5 % FBS and seeded on an XF24 microplate. The culture was incubated for 2 days, then the XF24 bioenergetic assay was initiated by removing the exhausted medium and replacing it with sodium-bicarbonate-free DMEM containing 5 % FBS. The extracellular flux changes in oxygen and in the pH of the medium surrounding the adherent cells were detected instantaneously by an XF24 extracellular Flux Analyzer (Seahorse Bioscience). The OCR and ECAR were detected at a steady state, then SSd (0.5 μM and 1 μM), oligomycin (1 μM), carbonyl cyanide 4-[trifluoromethoxy] phenylhydrazone (FCCP; 1 μM) and a mixture of rotenone (1 μM) and myxothiazol (1 μM) were injected sequentially into the wells to obtain the values of the maximal and non-mitochondrial respiration rate.

### Immunofluoresence staining

HSC-T6 cells were grown on coverslips in 24-well plates and incubated overnight. The cells were treated with SSd (1 μM) for 1 h, then stained with 100nM MitoTracker® Deep Red FM (Invitrogen™, Life Technologies) for 30 min at 37 °C. The cells were then fixed with 4 % cold paraformaldehyde for 20 min at 4 °C, and permeabilized with 0.1 % Triton X-100 for 1 min at room temperature. The cells were subsequently blocked with 1 % bovine serum albumin (BSA) for 30 min at room temperature and incubated with anti-Cyt c, anti-EndoG and anti-AIF antibody (GeneTex) overnight at 4 °C. The cells were then incubated with FITC-conjugated secondary antibody (Santa Cruz Biotechnology) for 60 min at room temperature, with DAPI (Molecular Probe) as a nuclear counterstain. The coverslips were mounted onto microscopy slides, and visualized under a confocal laser-scanning microscope (TCS SP5, LEICA).

### Statistical analyses

All data are shown as the means of 3 independent experiments (mean ± S.D.). Statistical analysis was performed by the unpaired Student’s *t*-test*,* with *p* < 0.01 as significant.

## Results

### SSd induced apoptosis, and reduced the protein expression of collagen I, collagen III and α-SMA in HSCs

**Fig. 1:**
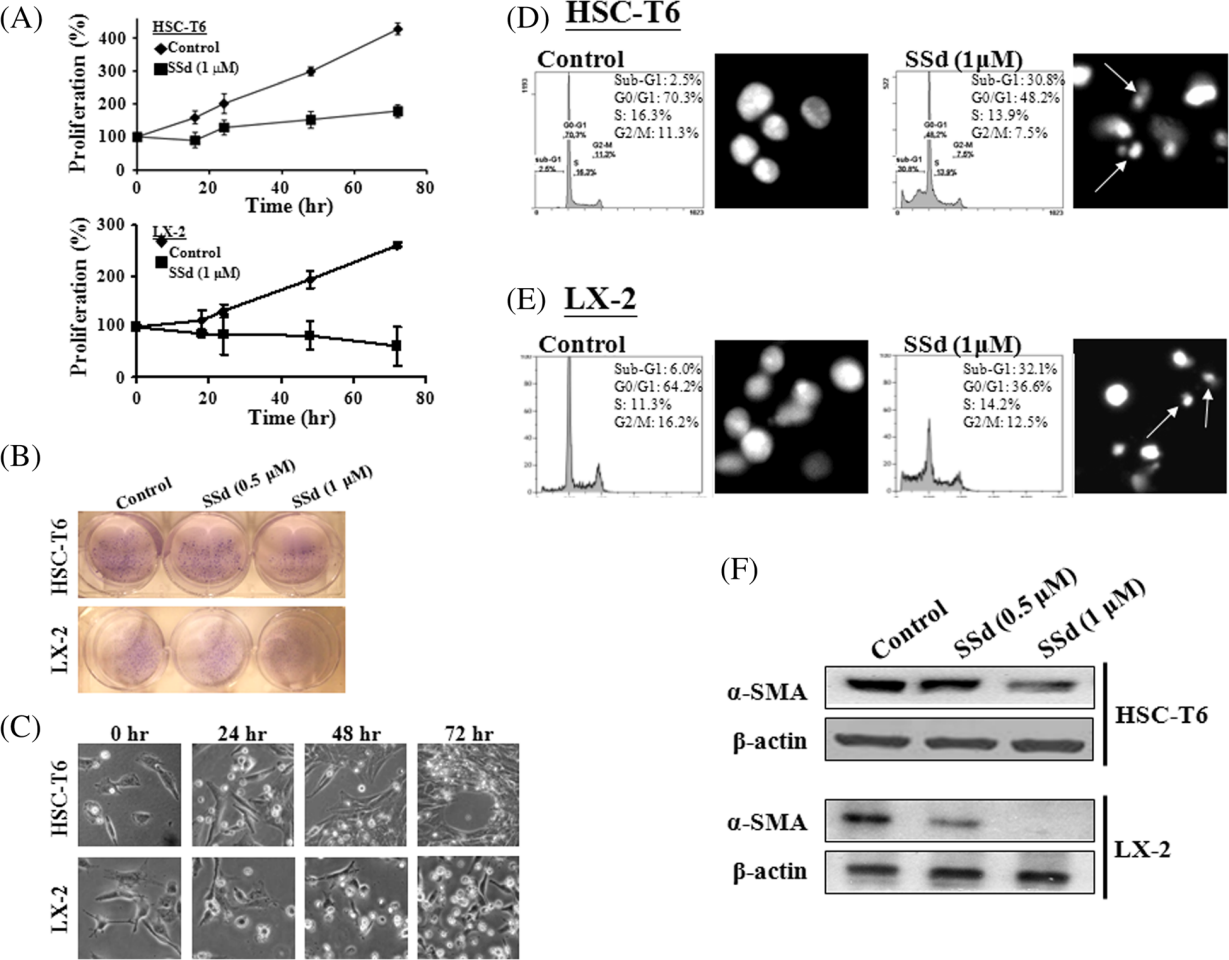
SSd inhibited cell proliferation and colony formation, induced apoptosis, and reduced α-SMA expression on HSC-T6 and LX-2 cells. (**a**) HSC-T6 and LX-2 cells were treated with SSd (1 μM) for 0, 16, 24, 48 and 72 h. Cell proliferation rate was detected by the MTT assay. (**b**) SSd inhibited HSCs cell growth as determined by colony formation assay. (**c**) Cell morphology was visualized by an optical microscope with magnification: 200×. Apoptotic cells of both HSC-T6 (**d**) and LX-2 cells (**e**) were detected by flow cytometry. (**f**) The α-SMA protein was measured in HSCs treated with a variety of SSd doses by Western blotting. Data are the mean ± S.D. from 3 independent experiments

**Fig. 2:**
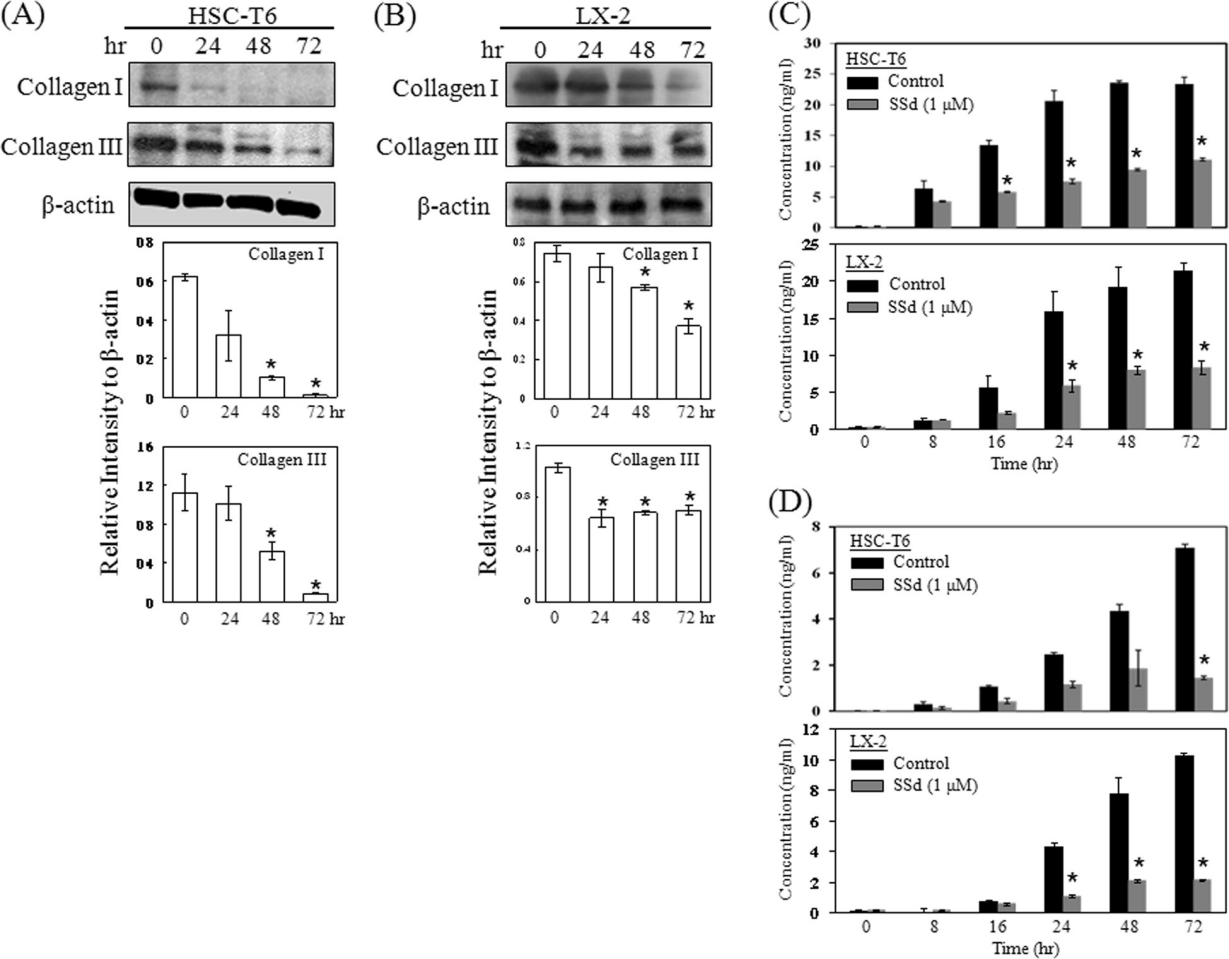
SSd inhibited the collagen I and III expression and secretion in HSC-T6 and LX-2 cells. (**a** and **b**) HSC-T6 and LX-2 cells were treated with or without SSd (1 μM) for 0, 24, 48 and 72 h. The total cellular protein content was extracted to measure the collagen type I and type III expression by western blotting. The fold change in protein expression is expressed as a ratio calculated by dividing the specific protein band density by the β-actin band density. The supernatant was collected to measure the secreted collagen type I (**c**) and collagen type III (**d**) by ELISA. *P < 0.01 versus time zero or the control group

To study the cytotoxic effects of SSd on HSCs of both HSC-T6 and LX-2, the MTT assay and colony formation assay were performed to examine the cell growth after exposure to SSd. SSd effectively inhibited cell proliferation of both HSC-T6 and LX-2 cells in a time-dependent manner (Fig. 1a). Additionally, SSd (0.5 μM and 1 μM) inhibited colony formation of both HSC-T6 and LX-2 cells (Fig. 1b). Numerous cell debris suddenly appeared when cells were treated with SSd (1 μM) at 72 h (Fig. 1c). Flow cytometry was analyzed to detect apoptotic signals (i.e., the sub-G1 phase of the cell cycle) after SSd treatment for 24 h. As indicated in Fig. 1d and e, the percentage of the sub-G1 phase and apoptotic bodies increased in both HSC-T6 and LX-2 cells treated with SSd. Activated HSCs are characterized by their expression of α-SMA and the synthesis of ECM constituents, particularly collagen I and III, during hepatic fibrosis [2, 21]. Our data indicate that SSd significantly reduced α-SMA expression in both HSC-T6 and LX-2 cells (Fig. 1f). Additionally, SSd markedly reduced collagen I and III expression within 72 h in both HSC-T6 and LX-2 cells by Western blotting and ELISA (Fig. 2a-d).

### The SSd-induced apoptotic effects on HSC-T6 and LX-2 cells were partially caspase-3-dependent

**Fig. 3:**
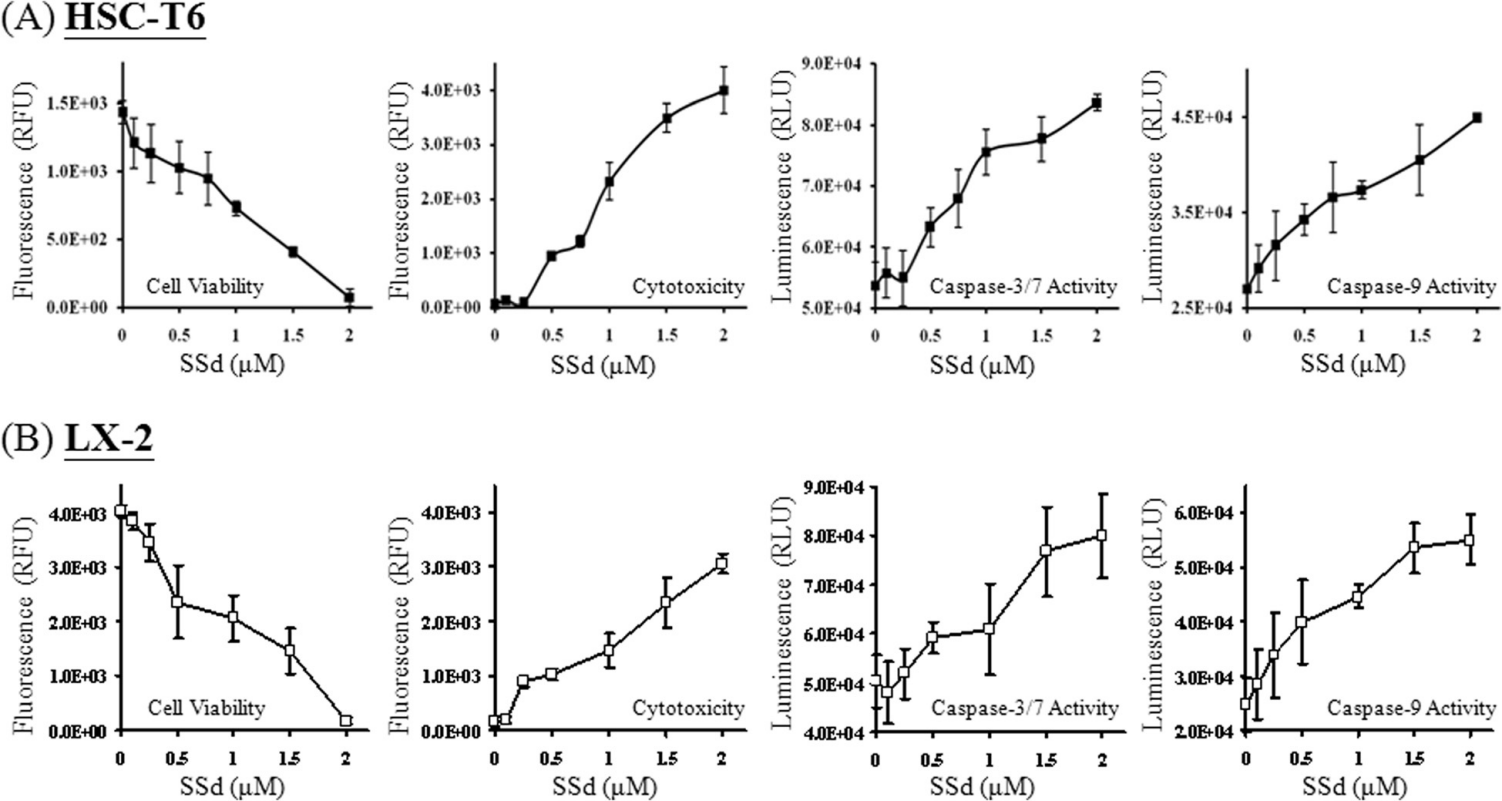
SSd induced cytotoxicity accompanied by an increase in caspase-3/7 and caspase-9 activity. (**a**) After treatment with serial concentrations of SSd for 24 h, cell survival of, cytotoxicity against, and caspase-3/7 activity in HSC-T6 cells were detected by an ApoTox-Glo™ Triplex assay kit. Caspase-9 activity was detected by a Caspase-Glo® 9 assay kit. (**b**) The same experiments described were performed in LX-2 cells. The data are the mean ± S.D. from 3 independent experiments

**Fig. 4:**
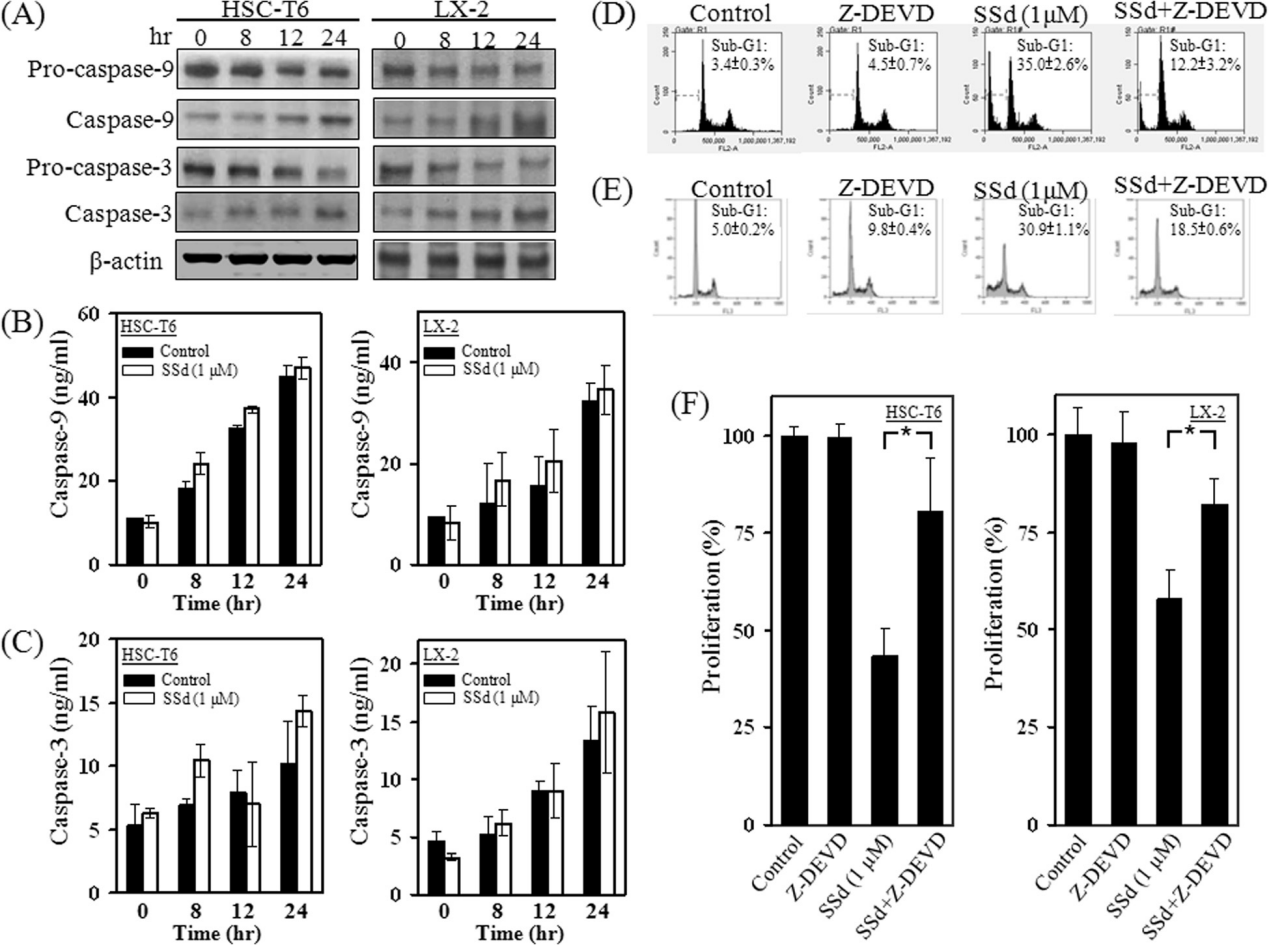
The SSd-induced apoptotic effects on HSC-T6 and LX-2 cells were partially caspase-3-dependent. (**a**) HSC-T6 and LX-2 cells were treated with SSd (1 μM) for 0, 8, 12 and 24 h, and the total cellular protein content was subsequently extracted to detect the pro-caspase-3, pro-caspase-9, caspase-3 and caspase-9 expression. The expression level of active caspase-3 and caspase-9 increased. (**b** and **c**) The protein expression level of caspase-3 and caspase-9 was also detected by ELISA kits. (**d** and **e**) HSC-T6 cells were treated with SSd (1 μM) for 24 h, and subsequently fixed by 70 % alcohol. The cells were stained with PI, and the cell cycle distribution was detected by flow cytometry. LX-2 cells were also treated with SSd (1 μM) for 24 h, and cell cycle distribution was detected by flow cytometry. The SSd-induced sub-G1 phase of HSC-T6 and LX-2 was partially inhibited by the caspase-3 inhibitor Z-DEVD-FMK. (**f**) SSd-induced cytotoxicity was detected by the MTT assay, and was also partially impeded by Z-DEVD-FMK. *P < 0.01

Caspase activity induction is involved in several ligand- and chemical-induced apoptotic processes. SSd treatment detected caspase-3/7 and caspase-9 activities in HSCs by using the ApoTox-Glo Triplex assay, Caspase-Glo 9 assay and Western blotting. The cytotoxicity, caspase-3/7 activity and caspase-9 activity of both HSC-T6 and LX-2 cells were increased, after treated with SSd for 24 h, as indicated in Fig. 3. Western blotting results also indicated that levels of caspase-3- and caspase-9-activated fragments rose after SSd treatment (Fig. 4a). To assess whether the total endogenous protein levels of caspase-3 and -9 were altered, the total forms of caspase-3 and -9 were measured by ELISA. Analytical results indicate that treatment with SSd for 24 h did not alter the total protein levels of caspase-3 and -9 compared to control groups in either HSC-T6 or LX-2 cells (Fig. 4b and c). A caspase-3 inhibitor, Z-DEVD-FMK, was adopted applied to investigate whether SSd-induced apoptosis was caspase-3-dependent. HSC-T6 and LX-2 cells were pre-incubated with Z-DEVD-FMK (100nM) for 1 h, and were subsequently co-treated with SSd for 24 h. Experimental results indicate that Z-DEVD-FMK partially inhibited the SSd-induced sub-G1 phase and cytotoxicity, as measured by flow cytometry and the MTT assay (Fig. 4d-f). These data indicate that SSd-induced apoptosis of both HSC-T6 and LX-2 cells may occur partially via caspase-3-dependent.

### SSd reduced ATP production, mitochondrial function and metabolism in HSC-T6 cells

**Fig. 5:**
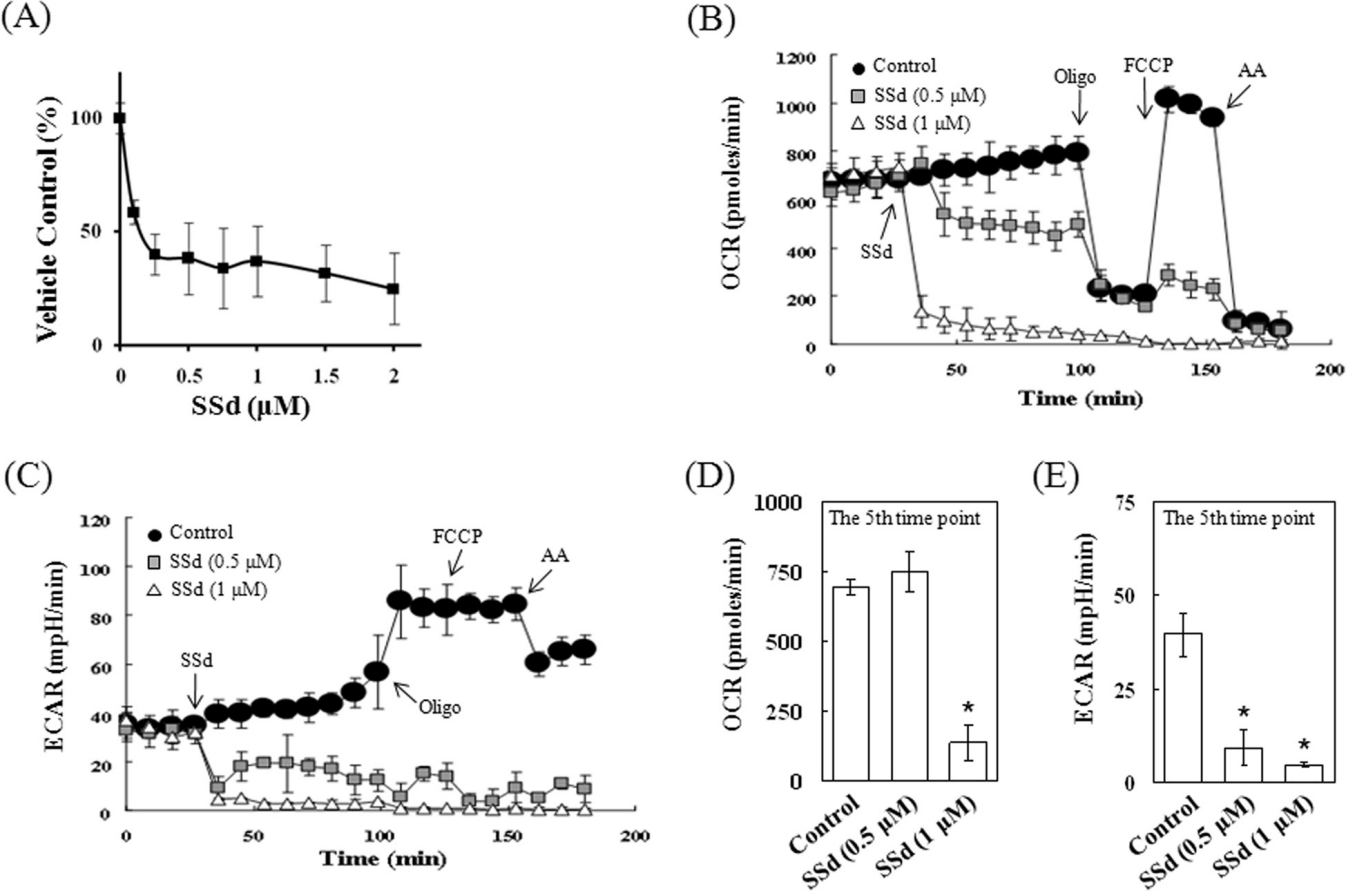
SSd blocked ATP production, mitochondrial oxygen consumption and extracellular acidification of HSC-T6 cells. (**a**) HSC-T6 cells were treated with a series of SSd concentrations, and the subsequent cellular ATP production was detected by the Mitochondrial ToxGlo assay. (**b** and **c**) The oxygen consumption rate (OCR) and extracellular acidification rate (ECAR) were detected by a Seahorse XF24 bioenergetic assay. The arrow points denote the injection of SSd, oligomycin (Oligo), FCCP and rotenone/myxothiazol (AA). Steady-state OCR and ECAR were measured before the SSd injection. Oligomycin was injected at the twelfth time point, while maximal oxygen consumption was measured at the fifteenth time point after FCCP injection. (**d** and **e**) The data of the 5^th^ time point of OCR and ECAR after SSd injection indicate that SSd (0.5 μM) had stronger inhibitory effects on ECAR than on OCR. The data are the mean ± S.D. from 3 independent experiments. *P < 0.01 versus the control group

Mitochondrial fracture is common in apoptotic processes, and results in apoptotic factor release and caspase-9 activation. An experiment was performed to measure ATP production in HSC-T6 cells using the Mitochondrial ToxGlo Assay. Treatment with the indicated concentrations of SSd reduced cellular ATP production (Fig. 5a). To assess SSd-induced changes in the cells’ metabolic capacity and extracellular acidification, the cellular oxygen consumption and extracellular acidification were measured simultaneously by the Seahorse XF24 system. The steady-state oxygen consumption and extracellular acidification were measured, and then SSd (0.5 μM or 1 μM) was injected into the system at the fourth time point to stimulate the cells. Oligomycin was then injected to inhibit ATP synthase, and FCCP was added by injection to assess the maximal oxygen consumption. Finally, a mixture of rotenone and myxothiazol was injected to confirm that the respiration changes were mainly due to altered mitochondrial respiration. Experimental data show that SSd (1 μM) reduced levels of OCR and ECAR significantly (Fig. 5b and c). Injection of 0.5 μM of SSd has no effect on OCR at the fifth time point after, but it significantly inhibited ECAR (Fig. 5d and e). These results may indicate that SSd reduces oxygen consumption and the mitochondrial metabolic capacity of HSC-T6 cells.

### SSd regulated pro-apoptotic and anti-apoptotic protein expressions, and changed the mitochondrial membrane potential, resulting in mitochondrial apoptotic factor release

**Fig. 6:**
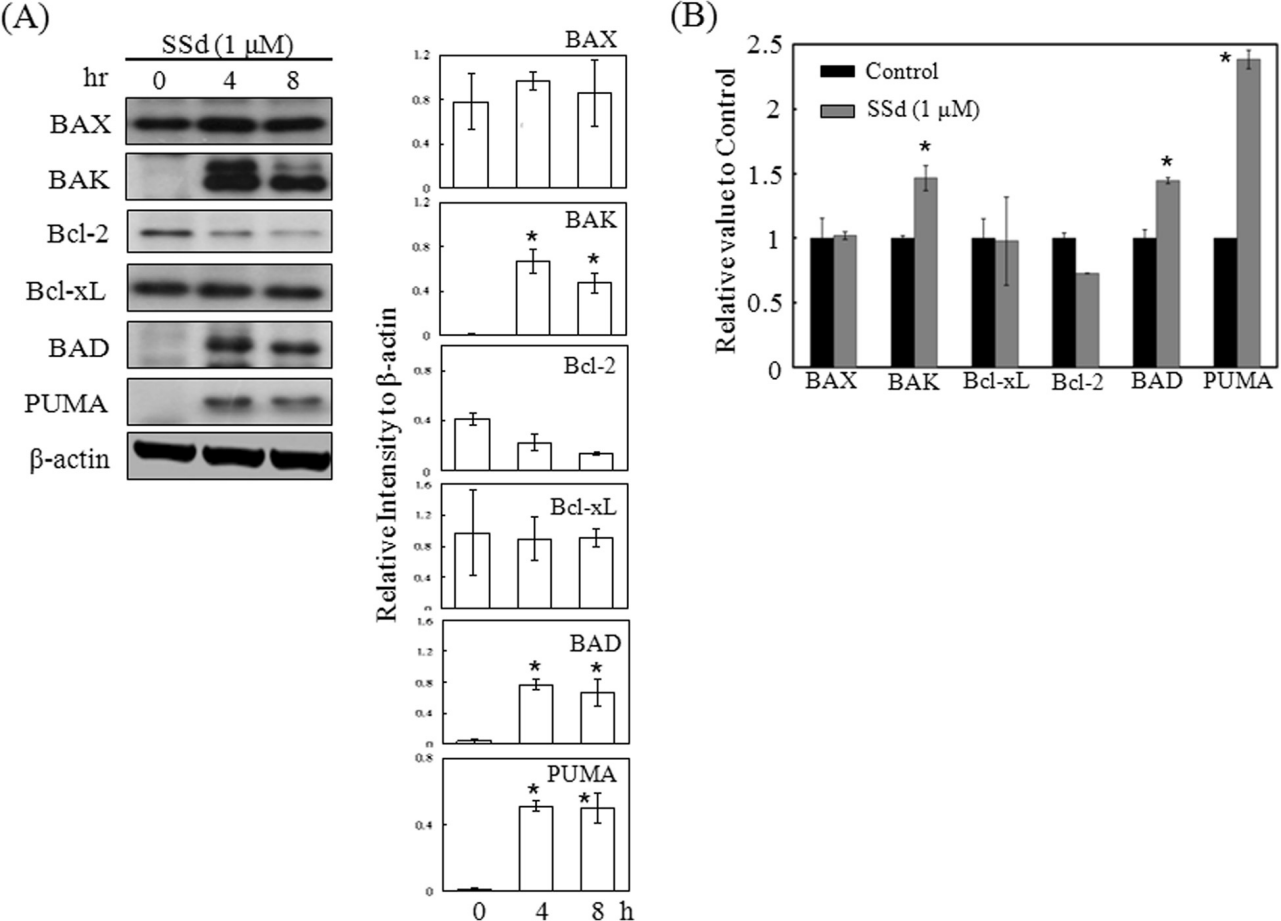
SSd reduced Bcl-2 expression, and increased BAK, BAD and PUMA expression. (**a**) HSC-T6 cells were treated with or without SSd (1 μM) for 0, 4 and 8 h. The total extracted protein content was analyzed by Western blotting to assess the protein expression of Bcl-2, Bcl-xL, BAX, BAK, BAD, and PUMA. (**b**) The total RNA of the HSC-T6 cells was extracted and quantified after treatment with or without SSd (1 μM) for 0 and 1 h. Reverse transcription PCR was performed with 3 μg of total RNA were used for. *Bcl-2*, *Bcl-xL*, *BAX*, *BAK*, *BAD*, *PUMA* and *GAPDH* cDNA were amplified and quantified using an ABI 7500 Real Time PCR System. *P < 0.01 versus the control group

**Fig. 7:**
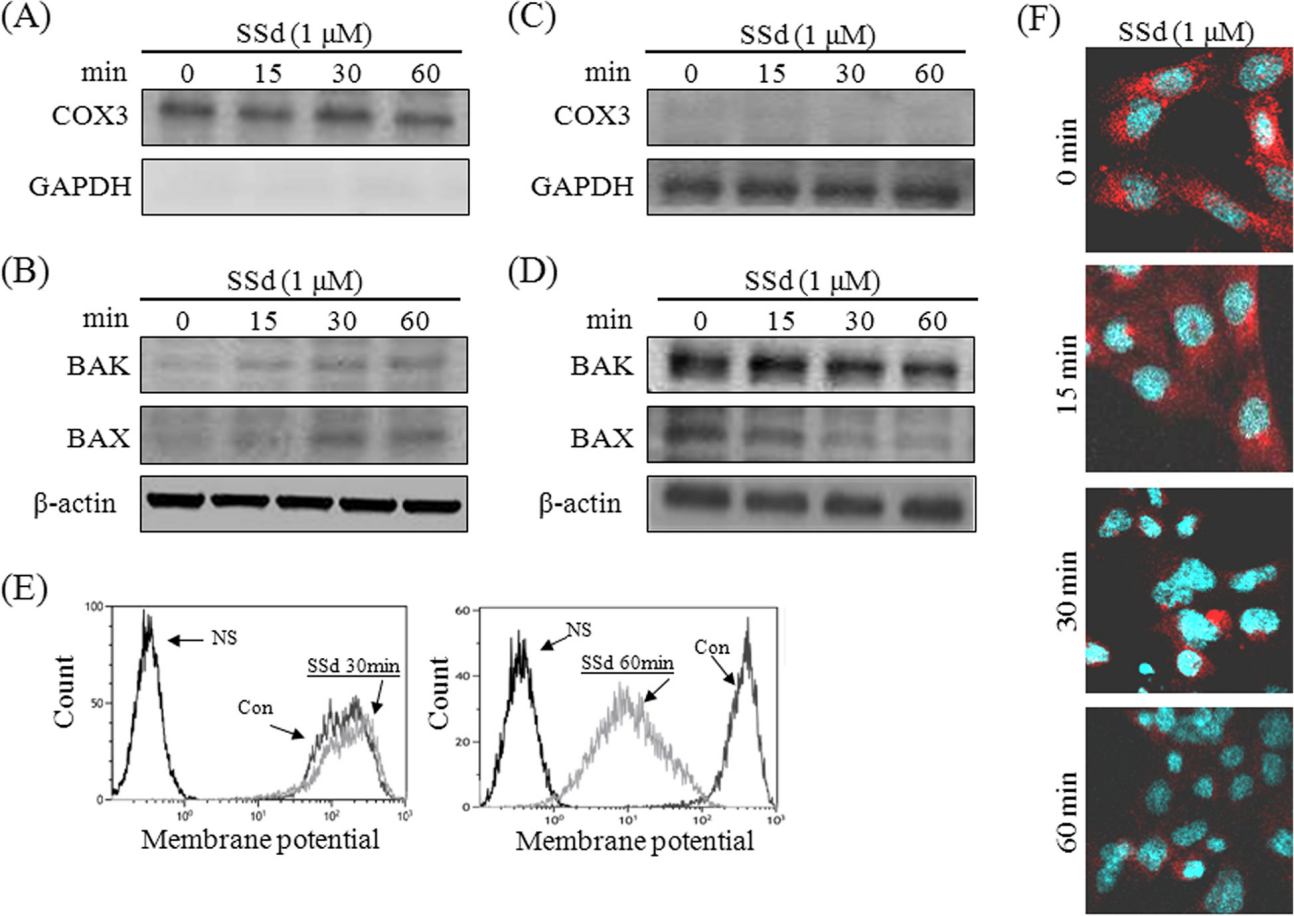
SSd triggered BAX and BAK translocation, and reduced the mitochondrial membrane potential. (**a**) HSC-T6 cells were treated with SSd (1 μM) for 0, 15, 30 and 60 min. The purity of mitochondrial fraction was validated by Western blotting with specific antibodies of mitochondria marker COX3 and cytosolic marker GAPDH. (**b**) SSd increased BAK and BAX expression in the mitochondrial fraction. (**c**) Cytosolic proteins were also applied to Western blotting. COX3 and GAPDH were also detected to validate the purity of the cytosolic fraction. (**d**) SSd reduced BAK and BAX expression in the cytosolic fraction. (**e**) The mitochondrial membrane potential (∆*ψm*) was monitored using a MitoProbe JC-1 assay kit, and was analyzed by flow cytometry. (**f**) HSC-T6 cells were grown in 24-well chamber cover glasses; treated with 1 μM SSd for 0, 15, 30 and 60 min, and analyzed using a confocal laser scanning microscope. Mitochondria were stained by the mitochondria-specific probe MitoTracker® Deep Red FM (100nM)

**Fig. 8:**
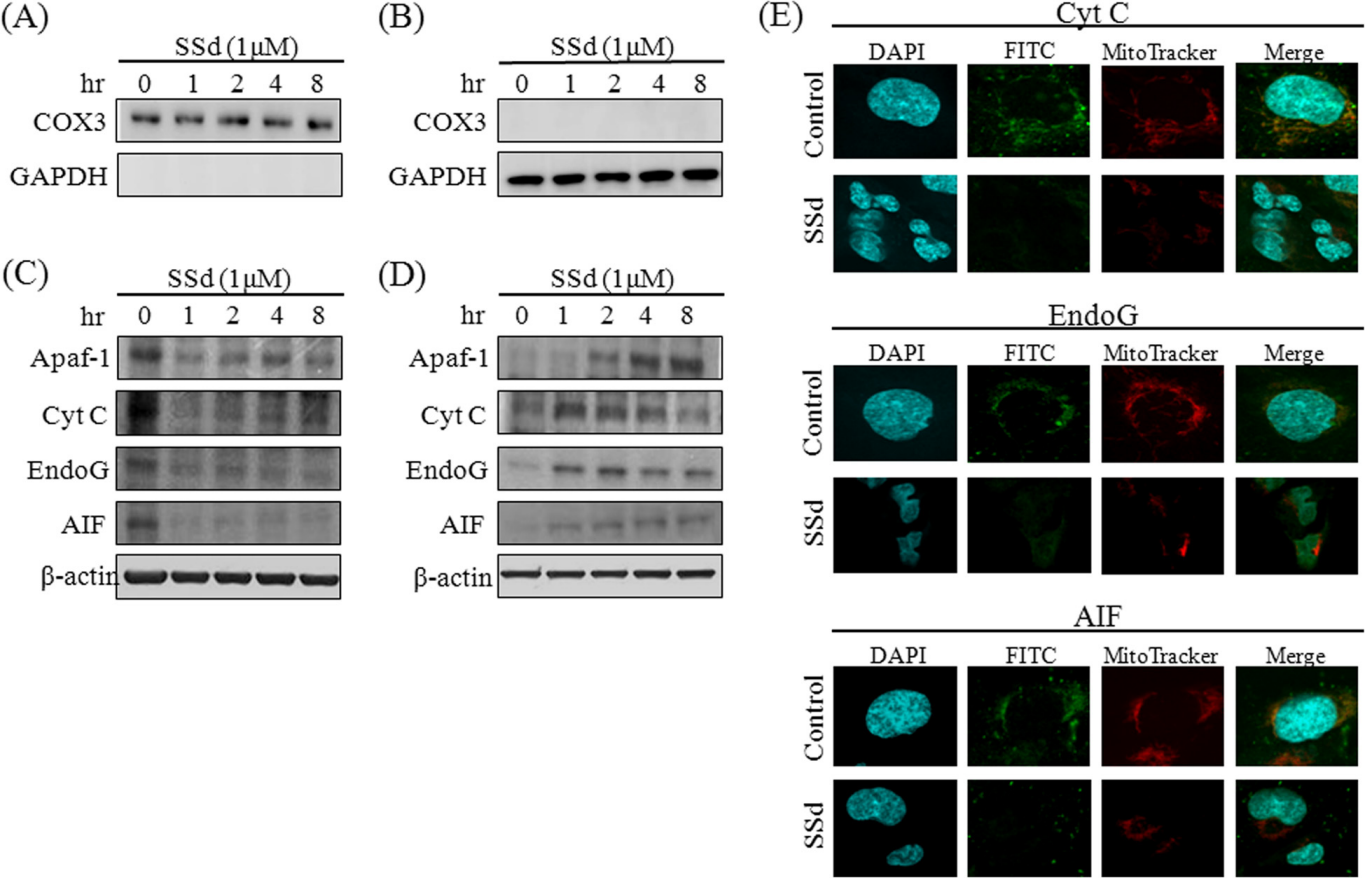
SSd triggered apoptotic factor release in HSC-T6 cells. The mitochondrial (**a**) and cytosolic (**b**) fractions were isolated following the treatment of HSC-T6 cells with 1 μM SSd. The purities of mitochondrial and cytosolic fraction were validated with anti-COX3 and anti-GAPDH antibodies by Western blotting. The expression levels of Apaf-1, Cyt c, EndoG and AIF were detected by Western blotting with specific antibodies in mitochondrial (**c**) and cytosolic (**d**) fractions. (**e**) HSC-T6 cells were grown in 24-well chamber cover glasses; treated with 1 μM SSd for 60 min; stained with MitoTracker® Deep Red FM (100 nM) for 30 min; fixed with 4 % cold paraformaldehyde, and incubated with specific primary antibodies and FITC-conjugated secondary antibody. DAPI was adopted as a nuclear counterstain. The fluorescence signals were analyzed by confocal laser scanning microscopy

After SSd treatment, the total protein content of HSC-T6 cells was extracted, and the expression of BAK, BAD, BAX, PUMA, Bcl-2 and Bcl-xL with specific antibodies was detected. SSd upregulated the BAK, BAD, and PUMA expressions within 8 h (Fig. 6a). Conversely, SSd downregulated the Bcl-2 expression, but did not affect the BAX or Bcl-xL expressions. The RNA expression levels of *BAX*, *BAD*, *Bcl-2*, and *PUMA* were consistent with the protein profiles (Fig. 6b). During apoptosis, BAX and BAK translocate from the cytoplasm to the mitochondria to form BAX/BAK pores. These BAX/BAK pores disturbed the membrane potential, leading to mitochondrial fracture and apoptotic factor release. To determine the effects experimentally, HSC-T6 cells were exposed to SSd (1 μM) exposure for 15, 30 and 60 min, and the mitochondrial and cytosolic fractions were then isolated for BAK and BAX detection. The organelle-specific marker COX3 expression was detected by Western blotting to ensure the purity of mitochondria [22]. As indicated in Fig. 7a, COX3 was present significantly in the mitochondrial fraction, while cytosol marker GAPDH was absent. BAK and BAX could be detected in the mitochondrial fraction of the 30- and 60-min SSd-treated cells (Fig. 7b). Additionally, GAPDH was present in the cytosolic fraction, but not in the mitochondrial fraction (Fig. 7c). SSd reduced BAK and BAX expressions in the cytosolic fraction within 60 min (Fig. 7d). The high purity of the mitochondria ensured that SSd increased BAK and BAX expression in mitochondria, while reducing it in cytoplasm. Moreover, the mitochondrial membrane potential and MitoTracker® Deep Red FM staining signal fell after SSd treatment (Fig. 7e and f). To further study the effect of SSd on apoptotic factor release, the mitochondrial and cytosolic fractions were isolated from HSC-T6 cells after SSd treatment. The purity of the mitochondrial and cytosolic fraction was also confirmed by the specific markers COX3 and GAPDH (Fig. 8a and b). Following SSd-induced mitochondrial function impairment, the mitochondial content of apoptotic factors, including Cyto c, EndoG, and AIF, fell while the cytoplasmic content of apoptotic factors rose (Fig. 8c and d). In addition, the apoptotic factor staining signal and mitochondrial staining signal fell after the 60-min SSd treatment, as revealed by fluorescent immunocytochemical staining and MitoTracker® Deep Red FM staining (Fig. 8e). These results suggest that SSd regulates pro- and anti-apoptotic protein expression and triggers BAX and BAK translocation, resulting in decrease of mitochondrial membrane potential, and apoptotic factor release.

## Discussion

The liver injury process may lead to HSC activation and high levels of α-SMA and collagen type I and III [2, 21]. Some previous studies have indicated that SSd protects liver function from CCl_4_- and dimethylnitrosamine-induced injury in rats [5, 8, 9]. These reports indicate that SSd-treated strategy for liver fibrosis may be safe, avoiding normal tissue injury. In our previous study, SSd inhibited HSC-T6 cell proliferation and migration. This study found that SSd induced apoptosis, and reduced α-SMA, collagen type I and collagen type III expression in HSC-T6 and LX-2 cells. Additionally, SSd-induced apoptosis was partially caspase-3 dependent. This is the first study to show that SSd-induced apoptosis on HSCs can be either caspase-3-dependent or caspase-3-independent. Moreover, our results also indicate that SSd triggers BAX/BAK translocation and apoptotic factor release. These data suggest that SSd inhibits HSCs activity and induces apoptosis. We conclude that SSd has potential for liver fibrosis treatment.

Mitochondria are essential cellular organelles that play a central role in ATP production and cell survival. However, mitochondria may also act as a regulator of the intracellular apoptotic pathway [23], and therefore have been considered as a potential target for chemotherapy. In this study, mitochondrial activity was estimated by a lumino- and XF24-bioenergetic assay. ATP production significantly fell after SSd treatment (Fig. 5a). Moreover, experimental data obtained by the XF24 bioenergetic assay indicate that SSd reduced the OCR of HSC-T6 cells at 0.5 μM, and almost completely inhibited it at a concentration of 1 μM (Fig. 5b). Oligomycin (an ATP synthase inhibitor) and FCCP (a proton ionophore) were injected into the cell culture microplate wells to assess the maximal OCRs. Finally, a mixture containing rotenone (an inhibitor of mitochondrial complex I) and myxothiazol (an electron transport blocker) was injected into the cell culture wells to confirm that the respiration changes resulted mainly from altered mitochondrial respiration. These data indicate that SSd significantly blocked the effect of FCCP (Fig. 5b) and might have inhibited ATP synthase, damaged the mitochondrial membrane and blocked the electron transport system in HSC-T6 cells. Extracellular acidification was detected simultaneously with oxygen consumption. The ECAR measurements reflected the metabolic activity of the HSC-T6 cells. Notably, SSd (0.5 μM) had a greater inhibitory effect on ECAR than on OCR (Fig. 5d and e). These data indicate that metabolism suppression may play a more important role in SSd-induced cell death and proliferation inhibition. A future study will investigate the underlying mechanism for this phenomenon.

Mitochondria-dependent apoptosis is regulated by the opposing actions of pro- and anti-apoptotic proteins of the Bcl-2 family, such as BAX and BAK that translocate from the cytosol to the mitochondrial outer membrane upon death signal stimulation. The translocated BAX and BAK subsequently form permeability transition pores, leading to apoptotic factor release and mitochondria rupture. Conversely, Bcl-2 and Bcl-xL, two anti-apoptotic proteins of the Bcl-2 family, inhibit BAX/BAK permeability transition pore formation and preserve mitochondrial integrity [24–26]. BAD is another pro-apoptotic protein of the Bcl-2 family involved in initiating apoptosis. It forms a heterodimer with Bcl-2 and Bcl-xL after activation, preventing them from arresting apoptosis [27]. Moreover, the pro-apoptotic Bcl-2 family protein PUMA is regulated by the tumor suppressor p53. After death signal stimulation, PUMA blocks the function of anti-apoptotic proteins, such as Bcl-2, Bcl-xL, Mcl-1 and Bcl-w, resulting in BAX/BAK translocation, apoptotic factor release, caspase activation and cell death [28]. The expression levels of pro- and anti-apoptotic proteins were measured to investigate mitochondria-dependent SSd-induced apoptosis. The levels of BAK, BAD, and PUMA increased, whereas that of Bcl-2 fell (Fig. 6).

Cyto c and Apaf-1, the main apoptotic factors, have essential roles in the mitochondria-dependent apoptotic pathway and trigger caspase activation in mammalian cells. Death signal stimulation causes the release of Cyto c and Apaf-1 from the mitochondria into the cytosol, leading to activation of caspase-9. Subsequently, pro-caspase-3 is converted to its active form (caspase-3) by caspase-9-mediated cleavage. Caspase-3 splits poly-ADP ribose polymerase (PARP) to cause DNA fragmentation and apoptosis [23]. Hence, the activation of caspase-3 can be considered as an important molecular marker for apoptosis. Moreover, mitochondria can also release AIF and EndoG to initiate caspase-3-independent apoptosis. During apoptotic signal stimulation, AIF and EndoG are released from the mitochondria, and translocate to the nucleus where they induce apoptosis by triggering chromatin condensation and DNA fragmentation [1, 29, 30]. This study found that SSd-induced apoptosis is partially caspase-3 dependent (Fig. 4). SSd also significantly reduced ATP production and mitochondrial function (Fig. 5). Additionally, BAX and BAK were detected in the mitochondrial fraction, while SSd reduced expressions of these two proteins in cytosolic fractions (Fig. 7b). Furthermore, the mitochondrial membrane potential and the MitoTracker signal declined in SSd-treated HSC-T6 cells (Fig. 7e and f). Finally, that the cytosolic protein fraction levels of Apaf-1, Cyt c, AIF, and EndoG increased (Fig. 8d). Taken together, these data indicate that SSd may trigger BAX and BAK translocation, resulting in a potential decrease in mitochondrial membrane levels, and release of apoptotic factors.

## Conclusion

In conclusion, SSd may induce caspase-3-dependent and independent apoptosis, which are mediated by mitochondrial dysfunction and fracture. Our work further reveals that Cyt c, Apaf-1, AIF, and EndoG may be involved in SSd-induced apoptosis.

## Abbreviations

HSCsHepatic stellate cells; SMA, Smooth muscle actin; SSd, Saikosaponin d
